# Enhancing curve and surface applications with trigonometric polynomial basis functions

**DOI:** 10.1371/journal.pone.0293970

**Published:** 2024-01-02

**Authors:** Aqsa Rasheed, Uzma Bashir, Farheen Ibraheem, Shazia Javed

**Affiliations:** 1 Department of Mathematics, Lahore College for Women University, Lahore, Pakistan; 2 Department of Mathematics, Forman Christian College University, Lahore, Pakistan; University of Hull, UNITED KINGDOM

## Abstract

This article primarily focuses on the utilization and importance of parametric curves in surface design. It delves into the construction and applications of parametric curves, exploring the implementation of trigonometric polynomial basis functions that possess two shape parameters. Initially, these basis functions are employed in constructing both rational and non-rational curves. Later, they are employed to define the surfaces generated by these curves. The discussion includes rational surfaces, tensor product surfaces, and various specialized surfaces. The aim is to provide a comprehensive understanding of the role and potential of parametric curves in surface design.

## 1 Introduction

Surfaces play a vital role in computer-aided geometric design (CAGD) with diverse applications. They are employed for tasks like fitting experimental data, handling tables of numbers, discretized solutions of differential equations, as well as designing various objects such as aircraft, cars, and other entities. Surfaces are also utilized in modeling human organs and robots [1]. The umbrella term for these applications, particularly in engineering, is known as computer-aided design/computer-aided manufacturing (CAD/CAM). It’s important to note that the choice of surface form depends on the specific application, as there isn’t a one-size-fits-all solution for all problems. CAGD has had a significant impact on fields like medical imaging, geographic information systems, computer gaming, and scientific visualization [2]. Furthermore, computer graphics stands out as one of the earliest and most significant applications of CAGD.

The CAGD is heavily reliant on curve and surface modeling. Computer-based machines and tools are a key contributor in the increased need for parametric curve-based surface modeling. These curves are not only useful for designing purposes but also useful in the path planning of robots. For example in the construction of 3D models of surfaces the path of the machine as well as the properties of desire objects are depending on these curves. Tool paths are curves that are controlled by user-based information in the form of algorithms. As a matter of fact, in the field of CAGD, surfaces based on parametric representation of curves are the most popular and suitable for building and modeling [3].

In CAGD Bézier curves are the most commonly used parametric curves due to their simple and user friendly nature. There are two main components in Bézier curve representation, basis functions and control points. Basis functions led to the generation of smooth continuous curves. while control points are responsible for the description of shape of the curve as Bézier curves mimics the shape of its control polygon.

While designing with traditional Bézier curves the shape of a curve and surface is fixed under a specific set of control points. For a slight change in the shape of the curve or surface one have to change the set of control points which may cause a huge change in the properties of curve or surface.

The problem at hand was to discuss the ways to design a curve or surface without altering the set of control points. This problem is addressed by many researchers purposing different polynomial basis functions which involves the shape parameters [4, 5]. Afterwards a new set of basis functions based on exponential functions is introduced [6]. Further different set of basis functions involving shape parameters and trigonometric functions for curve and surface design is introduced [7–10].

Later a generalization of Bézier like curves was introduced by extending the idea of shape parameters incorporated in generalized polynomial basis functions [11]. The idea of *n* − 1 shape parameters for an *n*th degree polynomial is drawn out [12, 13] for the description of curves and surfaces with an extra control. A generalization of Bézier like curves is given which include Timmer and Wang-Ball basis [14]. A form of generalized basis functions [15], which results in *k*th degree curve is elaborated for the purpose of curve modeling. The fractional Bézier curve [16] based on generalized fractional basis functions was next in queue to help designers to get a desired shape and design of the product.

Many authors have focused primarily on improving the forms of curves. The fundamental goal of this work, on the other hand, is to present and undertake a thorough investigation of the essential part that shape parameters play in the building of various types of surfaces. This construction is based on the development of a collection of blending functions for all degrees. A generalization of cubic trigonometric basis functions including two shape parameters has been provided for this purpose, allowing the user to construct curves and surfaces with more shape control.

Since surfaces are considered to be a fundamental component in numerous geometric modeling systems. Therefore, the main objective of this study is to offer the concept of surfaces formed by parametric curves, thereby expanding our understanding in this area.

## 2 Curve designing

Curves play a vital role as the fundamental building blocks for generating surfaces. When exploring the realm of curves, the most promising approach lies in utilizing the parametric representation of curves. Specifically, Bézier-like trigonometric curves are employed as a powerful tool for surface generation due to their control point form. In addition to control points and weight factors, shape parameters play a crucial role in rational and non-rational trigonometric Bézier curves as describing tools for surface generation.

### 2.1 Trigonometric polynomial functions with shape parameters

For *n* ≥ 4, the formulation for trigonometric polynomial functions with two shape parameters is as follow:

$$\begin{array}{c} B_{j}^{n}\left(\vartheta \right)=\{\begin{array}{cc} \left(1-\text{sin}\left(\vartheta \right)\right)B_{j}^{n-1}\left(\vartheta \right)+\text{sin}\left(\vartheta \right)B_{j-1}^{n-1}\left(\vartheta \right) & j=0,1,2,...,\lfloor n/2\rfloor -1\\ \text{sin}\left(\vartheta \right)B_{j-1}^{n-1}\left(\vartheta \right)+\text{cos}\left(\vartheta \right)B_{j}^{n-1}\left(\vartheta \right) & j=\lfloor n/2\rfloor \\ \left(1-\text{cos}\left(\vartheta \right)\right)B_{j-1}^{n-1}\left(\vartheta \right)+\text{cos}\left(\vartheta \right)B_{j}^{n-1}\left(\vartheta \right) & j=\lfloor n/2\rfloor +1,...,n-1,n \end{array} \end{array}$$ (1)

where $B_{j}^{n}\left(\vartheta \right)=0$ for *j* = −1 and *j* ≥ *n*. The formulation is based on degree three trigonometric polynomial functions with two shape parameters [9], which are defined as:

$$\begin{array}{c} \left\{ \begin{array}{c} B_{0}^{3}\left(\vartheta \right)={\left(1-\text{sin}\left(\vartheta \right)\right)}^{2}\left(1+\left(1-l_{1}\right)\text{sin}\left(\vartheta \right)\right)\\ B_{1}^{3}\left(\vartheta \right)=\text{sin}\left(\vartheta \right)\left(1-\text{sin}\left(\vartheta \right)\right)\left(\left(1+\text{sin}\left(\vartheta \right)\right)+l_{1}\left(1-\text{sin}\left(\vartheta \right)\right)\right)\\ B_{2}^{3}\left(\vartheta \right)=\text{cos}\left(\vartheta \right)\left(1-\text{cos}\left(\vartheta \right)\right)\left(\left(1+\text{cos}\left(\vartheta \right)\right)+l_{2}\left(1-\text{cos}\left(\vartheta \right)\right)\right)\\ B_{3}^{3}\left(\vartheta \right)={\left(1-\text{cos}\left(\vartheta \right)\right)}^{2}\left(1+\left(1-l_{2}\right)\text{cos}\left(\vartheta \right)\right) \end{array}\right. \end{array}$$ (2)

where *l*_1_, *l*_2_ ∈ [−1, 2] and *ϑ* ∈ [0, *π*/2].

Thees trigonometric polynomial functions $B_{j}^{n}\left(\vartheta \right)$, defined in Eqs 1 and 2 fulfill the favorable geometric properties:

1. non-negativity: $B_{j}^{n}\left(\vartheta \right)\geq 0$,
2. sum to unity: $\sum_{j=0}^{n}B_{j}^{n}\left(\vartheta \right)=1,\;j=0,1,2,...,n$,
3. symmetry: These basis functions are symmetric with respect to the parameters *ϑ* and $\left(\frac{\pi }{2}-\vartheta \right)$
  $$B_{j}^{n}\left(\vartheta ,l_{1},l_{2}\right)=B_{n-j}^{n}\left(\frac{\pi }{2}-\vartheta ,l_{2},l_{1}\right),\;j=0,1,2,...,n,$$
4. monotonicity: For the specified values of shape parameters *l* and *m*, the basis function $B_{0}^{n}\left(\vartheta \right)$ decreases and $B_{n}^{n}\left(\vartheta \right)$ increases monotonically.

**Fig 1:**
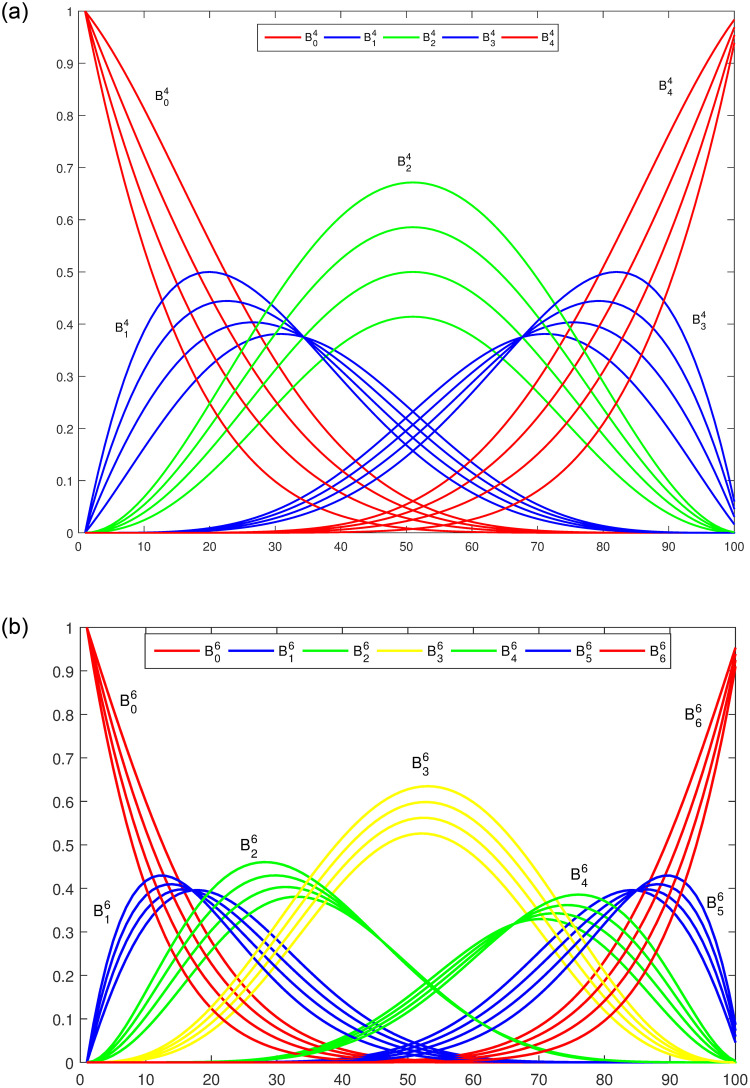
Trigonometric polynomial basis functions. (a) n = 4, *l*_1_ = *l*_2_ = −1, 0, 1, 2, (b) *n* = 6, *l*_1_ = _l__2_ = −1, 0, 1, 2.

The graphical illustrations exhibit the behavior of these basis functions with multiple shape parameters are shown in Fig 1. It is clear that all the basis functions are positive. In addition, first and last basis functions are monotonically decreasing and increasing respectively. These functions also satisfies the properties of symmetry.

### 2.2 Rational and non-rational parametric trigonometric Bézier curves

The polynomial trigonometric basis functions defined in Eq 1 play a crucial role in constructing parametric Bézier curves of degree *n* ≥ 3. These basis functions serve as the essential components that govern the shape and behavior of the curves. The parametric Bézier curves can be represented in the following control point form:

$$\begin{array}{c} C\left(\vartheta \right)=\sum_{j=0}^{n}Q_{k}B_{j}^{n}\left(\vartheta \right),\;\vartheta \in \left[0,\frac{\pi }{2}\right]. \end{array}$$ (3)

Here C(ϑ) represents the parametric Bézier curve, $Q_{k}$ denotes the control points and $B_{j}^{n}\left(\vartheta \right)$ represents the polynomial trigonometric basis functions. By adjusting the control points and incorporating the appropriate basis functions, we can manipulate and shape the parametric Bézier curves to achieve desired outcomes. The degree of the curve, denoted by *n*, determines the number of control points and the level of flexibility in the curve’s shape.

To enhance control over the shape of a curve, it is possible to assign positive weights $W_{j}$ to each control point. This approach gives rise to rational trigonometric Bézier curves, as defined in Eq 4. These curves extend the concept of parametric Bézier curves by incorporating weight factors for the control points. The rational trigonometric Bézier curve can be represented as follows:

$$\begin{array}{c} C\left(\vartheta \right)=\frac{\sum_{j=0}^{n}\left(\vartheta \right)W_{j}Q_{j}B_{j}^{n}}{\sum_{j=0}^{n}B_{j}^{n}\left(\vartheta \right)W_{j}},\;\vartheta \in \left[0,\frac{\pi }{2}\right]. \end{array}$$ (4)

In this formulation, C(ϑ) denotes the rational trigonometric Bézier curve, $Q_{j}$ represents the control points, $W_{j}$ signifies the assigned positive weights, and $B_{j}^{n}\left(\vartheta \right)$ represents the polynomial trigonometric basis functions. The weights provide additional control over the influence of each control point on the resulting curve shape, allowing for more flexibility and customization.

By adjusting the weights assigned to the control points, designers can fine-tune the behavior and shape of the rational trigonometric Bézier curves, catering to specific design requirements and achieving desired visual effects.

The following simplified form can be obtained from Eq 4,

$$\begin{array}{c} C\left(\vartheta \right)=\sum_{j=0}^{n}R_{j}^{n}\left(\vartheta \right)Q_{j},\;\vartheta \in \left[0,\frac{\pi }{2}\right], \end{array}$$ (5)

where

$$\begin{array}{c} R_{j}^{n}\left(\vartheta \right)=\frac{B_{j}^{n}\left(\vartheta \right)W_{j}}{\sum_{k=0}^{n}B_{k}^{n}\left(\vartheta \right)W_{k}},\;\vartheta \in \left[0,\frac{\pi }{2}\right]. \end{array}$$ (6)

### 2.3 Properties of rational and non rational parametric curves

1. Endpoint interpolation
  When considering the defined curve over the interval $\left[0,\frac{\pi }{2}\right]$, the following properties can be observed for the basis functions:
  For *ϑ* = 0, $B_{j}^{n}\left(0\right)=0$ for all *j* ≥ 1, and $B_{0}^{n}\left(0\right)=1$,

$\vartheta =\frac{\pi }{2}$ , $B_{j}^{n}\left(\frac{\pi }{2}\right)=0$ for all *j* ≤ *n* − 1, and $B_{n}^{n}\left(0\right)=1$.
  From Eq 3, it follows that the *nth* degree curve interpolates first and last control points. In addition, the first derivative of parametric curves at end points represents tangents vectors.
  $$C\left(0\right)=Q_{0},$$
  $$C\left(\frac{\pi }{2}\right)=Q_{n},$$
  $$C^{{}^{\prime }}\left(0\right)=\left(n-2+l_{1}\right)\left(Q_{1}-Q_{0}\right),$$
  $$C^{{}^{\prime }}\left(\frac{\pi }{2}\right)=\left(n-2+l_{2}\right)\left(Q_{n}-Q_{n-1}\right).$$
  For *nth* degree rational curve 4, the end points and tangents properties include the corresponding weights as well.
  $$C\left(0\right)=W_{0}Q_{0},$$
  $$C\left(\frac{\pi }{2}\right)=W_{n}Q_{n},$$
  $$C^{{}^{\prime }}\left(0\right)=\left(n-2+l_{1}\right)\frac{W_{1}}{W_{0}}\left(Q_{1}-Q_{0}\right),$$
  $$C^{{}^{\prime }}\left(\frac{\pi }{2}\right)=\left(n-2+l_{2}\right)\frac{W_{n}}{W_{n-1}}\left(Q_{n}-Q_{n-1}\right).$$
2. Convex hull
  The non-negativity of basis functions and positive weights ensures the curve to remain in the convex combination of basis and control points along with weights.
3. Geometric invariance
  The defined curve is geometrically invariant under affine transformation, translation and rotation due the the partition of unity of basis functions.
4. Symmetry
  The curve is symmetric if the order of control points is reversed.
5. Shape adjustable property
  The shape of the curve can be controlled using shape parameters as well as assigned weights of the curve.

**Fig 2:**
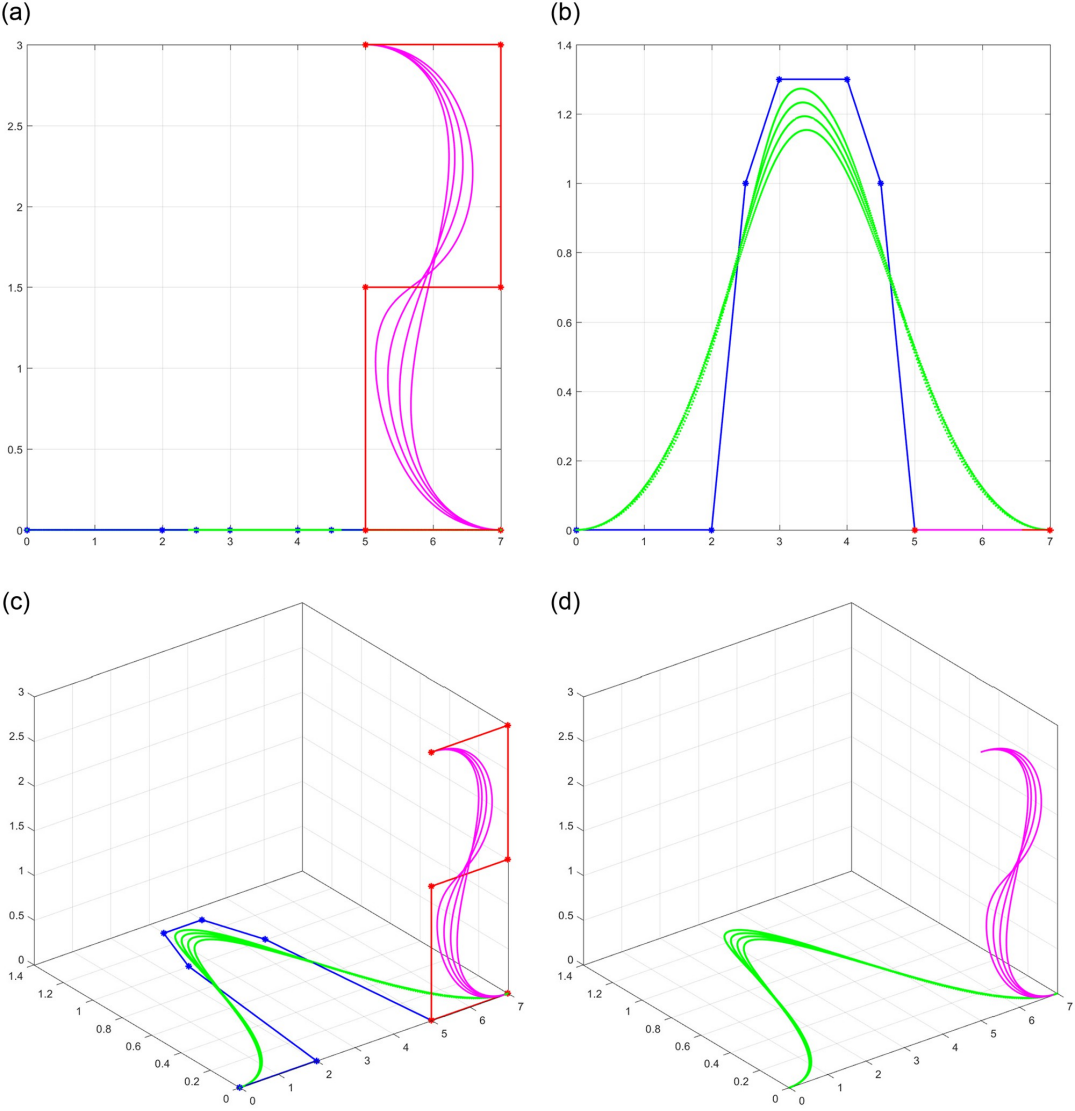
Behavior of shape parameters in non-rational form for *l*_1_ = *l*_2_ = −1, 0, 1, 2. (a) profile curve, (b) trajectory curve, (c) 3D view of curves along control polygon, (d) 3D view of curves.

**Fig 3:**
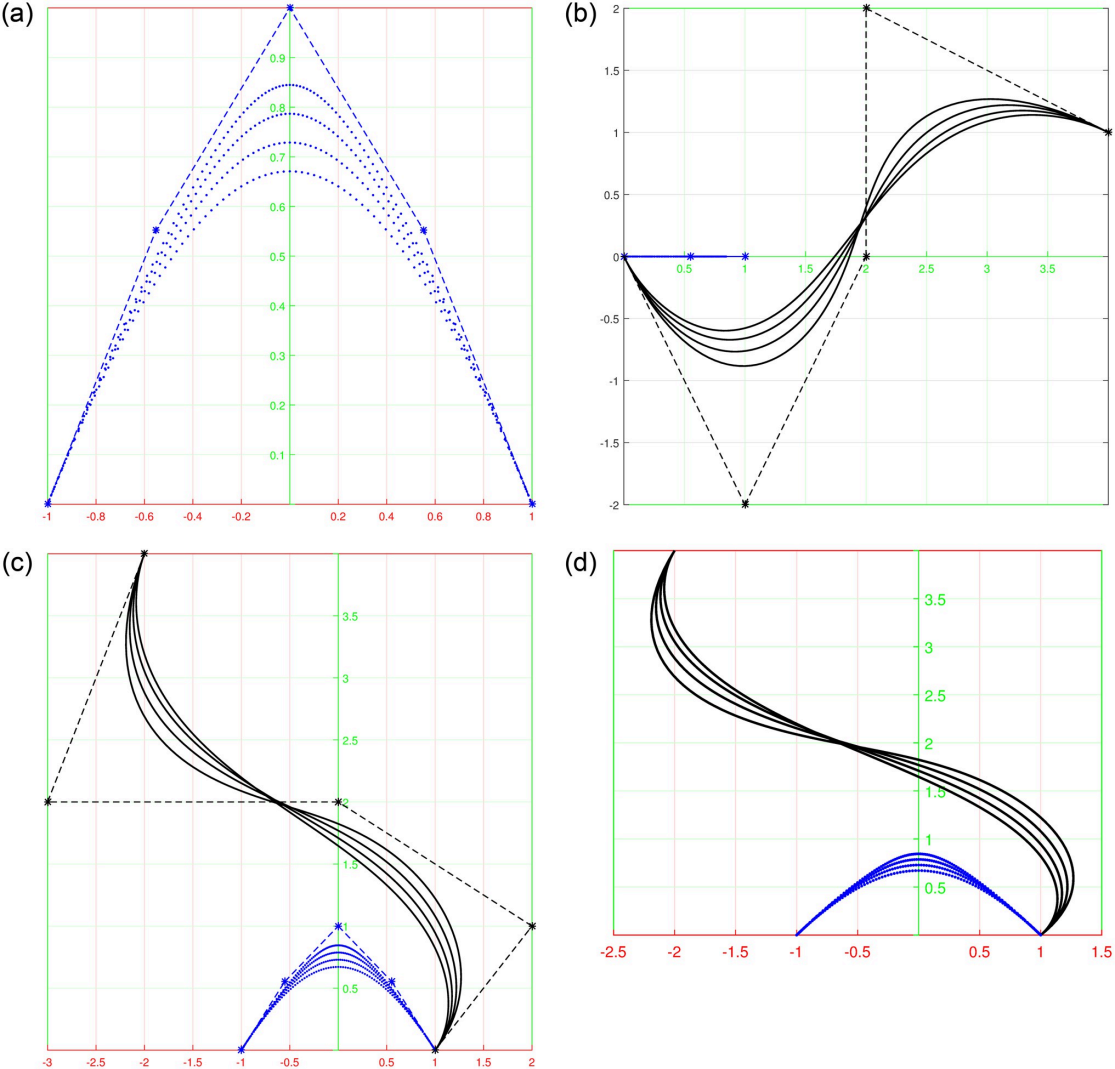
Behavior of shape parameters in rational form for *l*_1_ = *l*_2_ = −1, 0, 1, 2. (a) rational profile curve, (b) rational section curve, (c) 3D view of curves along control polygon, (d) 3D view of rational curves.

Shape parameters allow further customization of the curves by adjusting specific characteristics such as curve openness, convexity, or concavity. By manipulating shape parameters, one can achieve a wide range of curve variations, from smooth and flowing profiles to sharp and intricate profile and trajectory curves shown in Fig 2, or even dynamically changing section curves represented in Fig 3. The versatility offered by shape parameters enhances the expressive power of trigonometric Bézier curves, making them indispensable tools in the field of surface generation.

### 2.4 Advantages of the proposed trigonometric Bézier-like basis

Computer aided geometric design mainly relies on Bézier form for the construction and manipulation of curves and surfaces. The standard Bézier curves possess remarkable properties, and their significance is unquestionable. Therefore, the natural question arises that why there is the need of new set of functions. Whether they are truly necessary and/or is there a meaningful space for their utilization?

There are many reasons, few of which are listed below, that support the idea of introducing new set of functions in different domains. Trigonometric basis functions are one of those emerging functions. Beyond their well-established real-life applications, there are numerous areas where trigonometric-based Bézier-like curves can excel. However, introducing the concept of trigonometric polynomials should not be perceived as a challenge to the importance of standard Bézier curves.

In this era of machine learning and artificial intelligence, the need for new basis functions becomes even more vital. Some other notable examples are:

1. Computer Graphics and Animation:
  Trigonometric Bézier curves are well-suited for modeling circular and elliptical shapes in computer graphics and animation. They can be used to create smooth arcs and curves in characters, objects, or paths of motion and can also be used to design fonts. Trigonometric Bézier curves can be used in physics simulations to model circular or elliptical motions [17], as they naturally describe these trajectories.
2. AI-Based Generative Design:
  AI algorithms that generate art can use trigonometric Bézier curves to create aesthetically pleasing, curvilinear designs. In digital art and sculpting applications, artists may use trigonometric Bézier curves to create unique and complex shapes with precise control.
3. Gesture Recognition:
  Trigonometric Bézier curves can be used in AI systems for recognizing and replicating handwritten or gestural input, where natural curves are important.
4. Motion Planning and Robotics
  In robotics and autonomous systems, trigonometric Bézier curves can be used for path planning [18], especially when you need robots to follow circular or curved trajectories [19].
5. Signal Processing:
  Trigonometric functions can be used to model complex wave forms for audio synthesis and signal processing, which can be applied in AI-driven music composition and sound design.
6. Image Compression:
  Trigonometric Bézier curves can be employed effectively in image compression techniques, offering unique advantages and capabilities compared to traditional methods [20]. These curves can address some of the limitations and challenges associated with traditional image compression methods, providing enhanced image quality, reduced artifacts, and improved scalability for various applications.
7. Machine Learning for Pattern Recognition:
  In certain pattern recognition tasks, trigonometric Bézier curves can be part of feature extraction techniques when dealing with curved patterns in images or data. Trigonometric-based curves may find applications in cognitive modeling and neuroscientific research, where they can be employed to simulate brain activity patterns and behavior more accurately.
8. Geometric Approximations:
  Trigonometric polynomials can provide superior approximations for certain complex geometries, which can be useful in fields like computer graphics and geometric modeling.

In summary, while standard Bézier curves have their undeniable merits, the introduction of trigonometric-based alternatives should be viewed as a complementary approach to meet the evolving demands of AI-driven design and various specialized domains. These new functions offer unique advantages and capabilities that can make a substantial difference in specific applications, where precision, expressiveness, and noise reduction are critical factors.

## 3 Construction of generalized trigonometric Bézier surfaces

A curve is a vector valued function with one parameter, whereas a surface is a vector valued function with two parameters. A curve is also known as a deformation or mapping of a straight line, on the other hand a surface is a mapping of a region R, of the uv–plane into Euclidean three dimensional geometry. Therefore it has the form S(u,v)=(x(u,v),y(u,v),z(u,v)),(u,v)∈R.

The polynomial trigonometric functions also serves as a major part for designing surfaces. Surface generation is another useful application of these curves. This section discusses ways to construct a surface using parametric trigonometric Bézier curves.

### 3.1 Rational trigonometric Bézier surfaces

In rational trigonometric Bézier surfaces, non negative weights are associated with each control point. These weights determine the influence of control points on the shape of the surface. The (*n* + 1) × (*m* + 1) weights correspond to the grid of control points used to define the surface. By adjusting these weights, designers can control the smoothness and deformation in different regions of the surface. Thus, these weights provide precise control over the surface’s behavior locally. This approach streamlines the design process, preserves the structural integrity of the surface, and offers real-time flexibility for creative exploration and fine-tuning.

A rational trigonometric Bézier surface of degree (*n*, *m*) with $Q_{k}$ control points and positive weights $W_{j,k}$ is defined as

$$\begin{array}{c} S^{n,m}\left(u,v\right)=\frac{\sum_{j=0}^{n}\sum_{k=0}^{m}B_{j}^{n}\left(u\right)B_{k}^{m}\left(v\right)Q_{j,k}W_{j,k}}{\sum_{j=0}^{n}\sum_{k=0}^{m}B_{j}^{n}\left(u\right)B_{k}^{m}\left(v\right)W_{j,k}},\;u,v\in \left[0,\frac{\pi }{2}\right], \end{array}$$ (7)

where $B_{j}^{n}\left(\vartheta \right)$ represents the polynomial trigonometric basis functions defined in Eq 1.

### 3.2 Tensor product patches

Non-rational Bézier surfaces are created by combining a bidirectional net of control points and the products of univariate Bernstein polynomials. The combination of two curves known as the tensor product surface defines a trigonometric Bézier surface of degree (n,m). The tensor product scheme based on parametric representation of surfaces, is perhaps the simplest and most extensively used approach in geometric modeling applications. It employs basis functions as well as geometric coefficients and can be written as:

$$\begin{array}{c} S^{n,m}\left(u,v\right)=\sum_{j=0}^{n}\sum_{k=0}^{m}B_{j}^{n}\left(u\right)B_{k}^{m}\left(v\right)Q_{j,k},\;u,v\in \left[0,\frac{\pi }{2},\right] \end{array}$$ (8)

by taking unit weights in 7.

### 3.3 Properties of trigonometric Bézier surfaces

Bézier patches exhibit numerous properties that are essentially analogous to those of Bézier curves. The defined surfaces 7 and 8 holds following properties listed below.

(i) Non-negativity:

$B_{j}^{n}\left(u\right)B_{k}^{m}\left(v\right)\geq 0$ for all $u,v\in [0,\frac{\pi }{2}]$.
(ii) Partition of unity:

$\sum_{j=0}^{n}\sum_{k=0}^{m}B_{j}^{n}\left(u\right)B_{k}^{m}\left(v\right)=1$ for all $u,v\in [0,\frac{\pi }{2}]$.
(iii) End point Interpolation:
  Similar to the Bézier curves, the Bézier surface patch can interpolate four boundary control points.
  $$S^{n,m}\left(0,0\right)=Q_{0,0},\;S^{n,m}\left(1,0\right)=Q_{n,0},$$
  $$S^{n,m}\left(0,1\right)=Q_{0,m},\;S^{n,m}\left(1,1\right)=Q_{n,m}.$$
  The control points along the control polygon boundaries serve as the control points for the boundary curves of the patch.
(iv) Affine invariant:
  Bézier surfaces exhibit affine invariance, meaning that they remain within the same class of surfaces under affine transformations, including translation, rotation, scaling, and shearing.
(v) Convex hull property:
  The surface $S^{n,m}\left(u,v\right)$ formed by a Bézier curve or surface lies within the convex hull of its control points. This means that the surface does not extend beyond the boundary formed by the control points.
(vi) Local control:
  Changes to individual control points primarily affect the portion of the surface near those points. This local control property allows for precise manipulation of the surface.
(vii) Symmetry:
  By re-indexing the control net, mainly taking any corner of control polygon as $Q_{0,0}$, ensures that the evaluation yields a patch with an identical shape to the original one.

### 3.4 Advanced surface construction techniques

Surfaces are characterized as the continuous movement of one or more curves. This section focuses on commonly used surfaces for manufacturing purposes. The construction of swung and swept surfaces is discussed. The main idea behind these techniques is to take one or two curves, or sets of curves, and generate a tensor product surface that smoothly connects these curves [3]. In other words, the given curves serve as isoparametric curves within the surface.

#### 3.4.1 Swung surfaces

The simplest way to understand the Swung surfaces is their description as a generalization of surface of revolution. Consider the profile curve P(u) of degree *n* using the Eq 5,

$$\begin{array}{c} P\left(u\right)=\sum_{j=0}^{n}R_{j}^{n}\left(u\right)Q_{j} \end{array}$$ (9)

defined in the *xz*–plane, and also let

$$\begin{array}{c} T\left(v\right)=\sum_{k=0}^{m}R_{k}^{m}\left(v\right)D_{k} \end{array}$$ (10)

be a trajectory curve in the *xy*–plane. Let the non-zero components of P(u) and T(v) are $P_{x}\left(u\right),P_{z}\left(u\right),T_{x}\left(v\right),$ and $T_{y}\left(v\right)$, then swung surface can be defined as

$$\begin{array}{c} S\left(u,v\right)=(\gamma P_{x}\left(u\right)T_{x}\left(v\right),\gamma P_{x}\left(u\right)T_{y}\left(v\right),P_{z}\left(u\right)). \end{array}$$ (11)

Geometrically S(u,v) is obtained when P(u) is swung about the *z* − *axis* and at the same time scaling it by a factor of *γ* and T(v). The invariance under transformation of these curves gives the representation of S(u,v) as

$$\begin{array}{c} S\left(u,v\right)=\sum_{j=0}^{n}\sum_{k=0}^{m}R_{j,k}^{n,m}\left(u,v\right)P_{j,k}, \end{array}$$ (12)

where

$$\begin{array}{c} P_{j,k}=Q_{j}D_{k}=\left(\gamma P_{j,x}T_{k,x},\gamma P_{j,x}T_{k,y},P_{j,z}\right), \end{array}$$

and

$$\begin{array}{c} W_{j,k}=W_{j}W_{k}. \end{array}$$

Both of the curves P(u) and T(v) can either be open or closed, giving an open or closed surface in both directions respectively.

#### 3.4.2 Swept Surfaces

Swept surfaces are generated by the movement of a single curve along a curved path. The curve can be moved along a route described by another curve. Let the route of the sweeping curve can be represented by R(v) and the cross section curve is defined by C(u). Then a swept surface can be define as [3],

$$\begin{array}{c} S(u,v)=R(v)+M(v)C(u), \end{array}$$ (13)

where M(v) is a 3-by-3 matrix of rotation and a factor for nonuniform scaling of C(u) based on v. C(u) and R(v) can be arbitrary curves of any degree. Swept surfaces can be classified into two types based on the rotation matrix. Here only the special type of the surface with identity unit rotation matrix is discussed.

## 4 Results

### 4.1 Rational trigonometric surface patch

**Fig 4:**
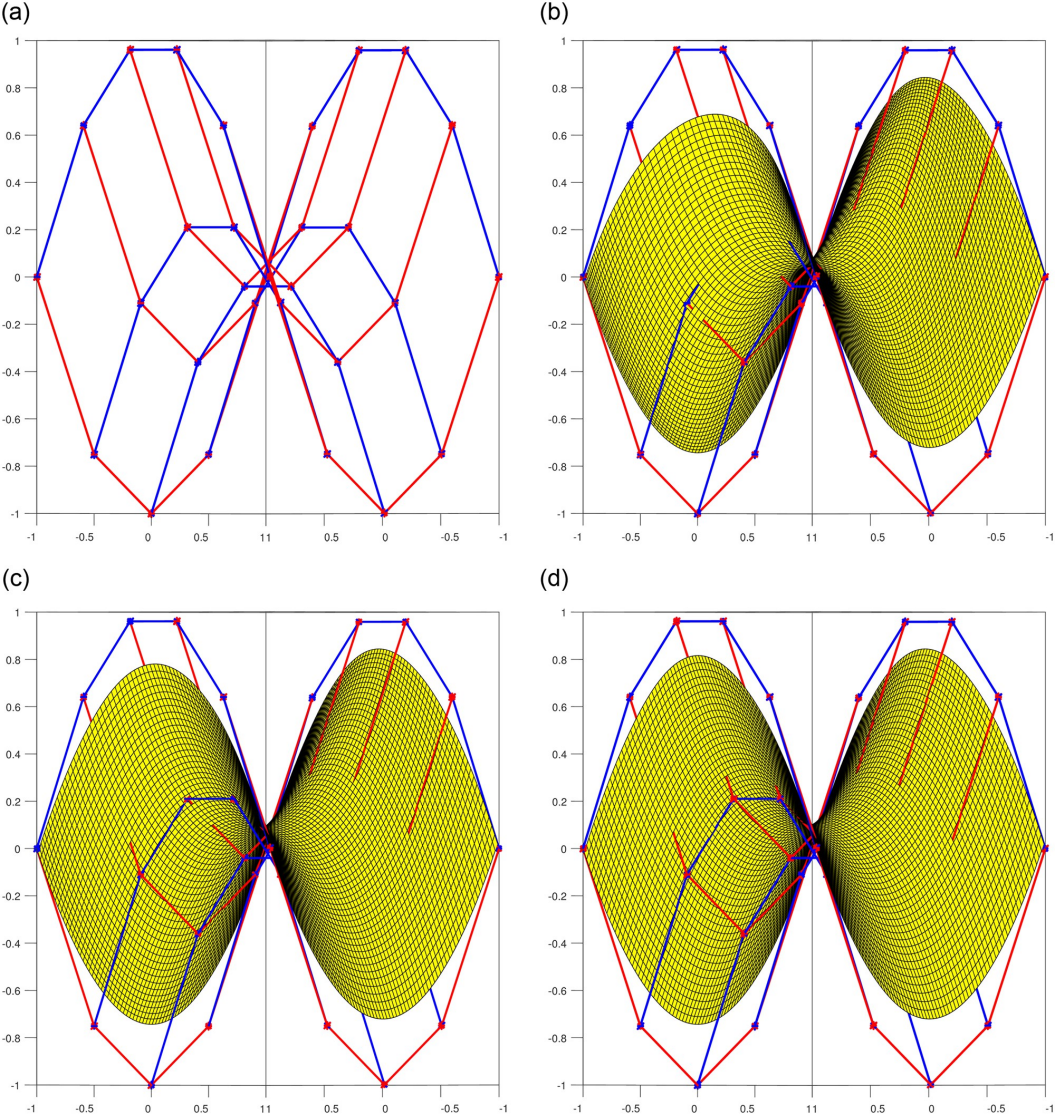
Rational quartic-quintic surface. (a) Qaurtic-quintic control net, (b) Central weights adjusted to 0.8, (c) Central weights adjusted to 0.5, (d) Central weights adjusted to 0.2.

In this illustration, a rational trigonometric surface patch is defined by taking *n* = 4 and *m* = 5. Within this framework, an approach is applied by adjusting the weights assigned to control points while keeping the shape parameters fixed. Despite utilizing the same control net, a user can create multiple distinct surfaces, three of them are shown in Fig 4. These surfaces are characterized by different middle weights while keeping the boundary weights fixed at unity.

### 4.2 Bi-Tensor product patches

**Fig 5:**
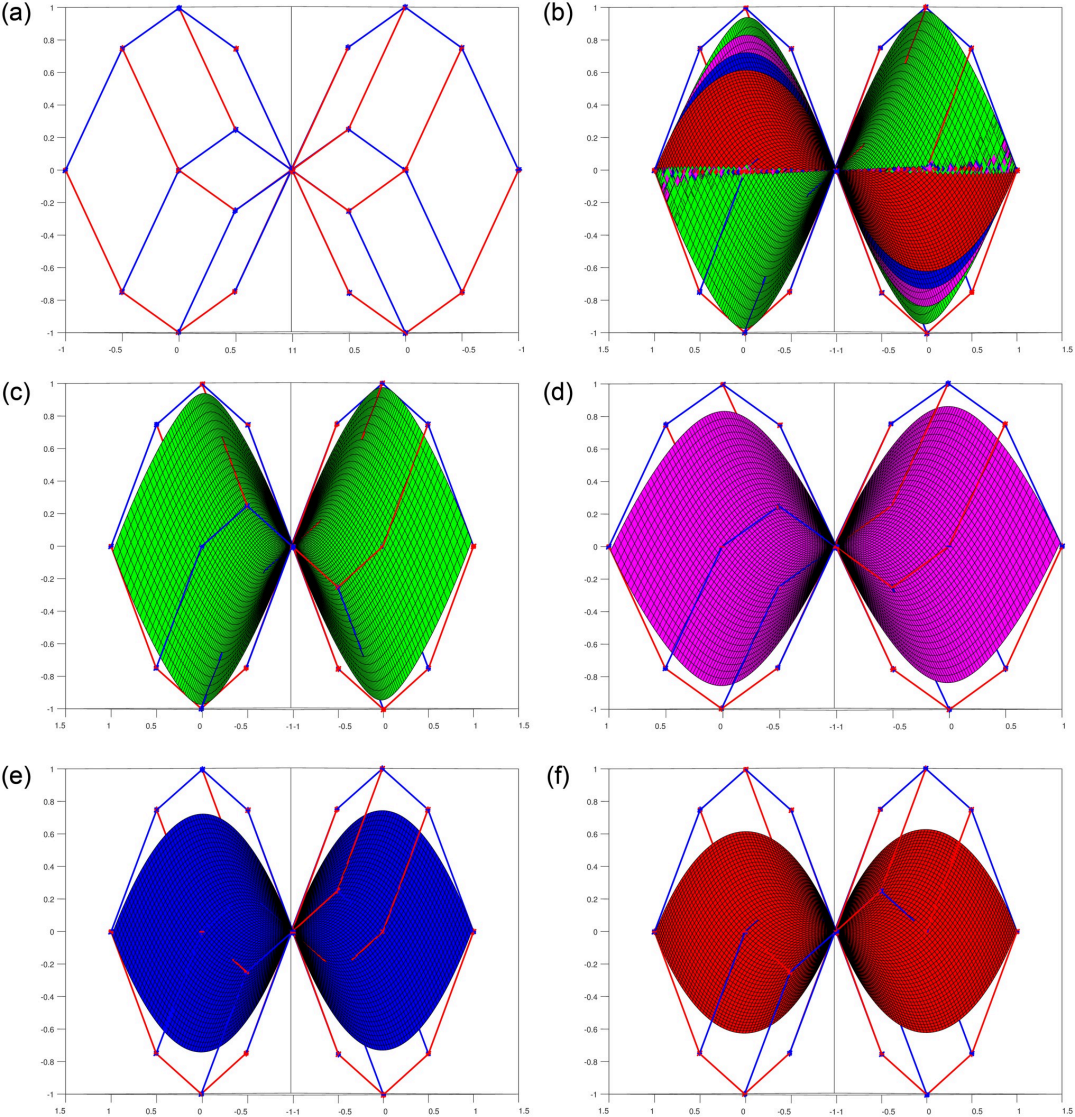
Bi-Quartic trigonometric patch with multiple shape parameters. (a) Bi-Qaurtic control net, (b) Corresponding four surfaces, (c) Bi-Quartic surface l1 = l2 = −1, (d) Bi-Quartic surface *l*_1_ = *l*_2_ = 0, (e) Bi-Quartic surface *l*_1_ = *l*_2_ = 1, (f) Bi-Quartic surface *l*_1_ = *l*_2_ = 2.

**Fig 6:**
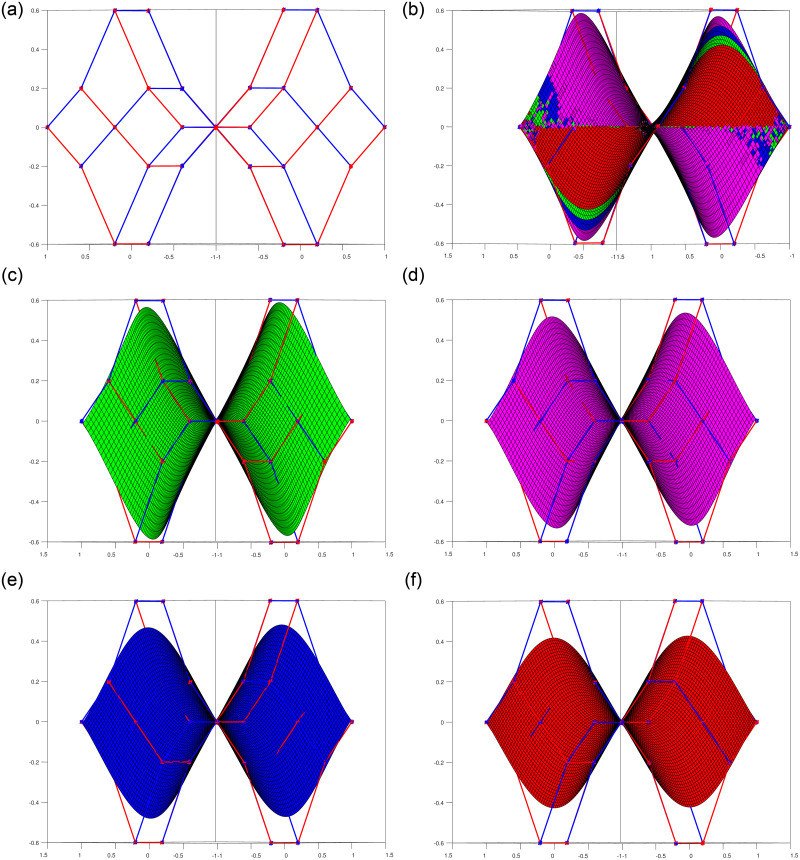
Bi-Quintic trigonometric patch with multiple shape parameters. (a) Bi-Quintic control net, (b) Corresponding four surfaces, (c) Bi-Quintic surface *l*_1_ = *l*_2_ = −1, (d) Bi-Quintic surface *l*_1_ = *l*_2_ = 0, (e) Bi-Quintic surface *l*_1_ = *l*_2_ = 1, (f) Bi-Quintic surface *l*_1_ = *l*_2_ = 2.

The most prevalent and advantageous approach to surface design is through tensor product surfaces. Tensor product patches are obtained by combining two curves of degree *n* and *m*. When *n* = *m*, we obtain Bi-tensor product patches. For example, when *n* = *m* = 4, the resulting surface is referred to as Bi-quartic shown in Fig 5, and for *n* = *m* = 5, it is known as Bi-quintic shown in Fig 6. The figures below depict both of these degree 4 and 5 surfaces and demonstrate the influence of shape parameters in constructing tensor product patches.

### 4.3 Open swung surface

**Fig 7:**
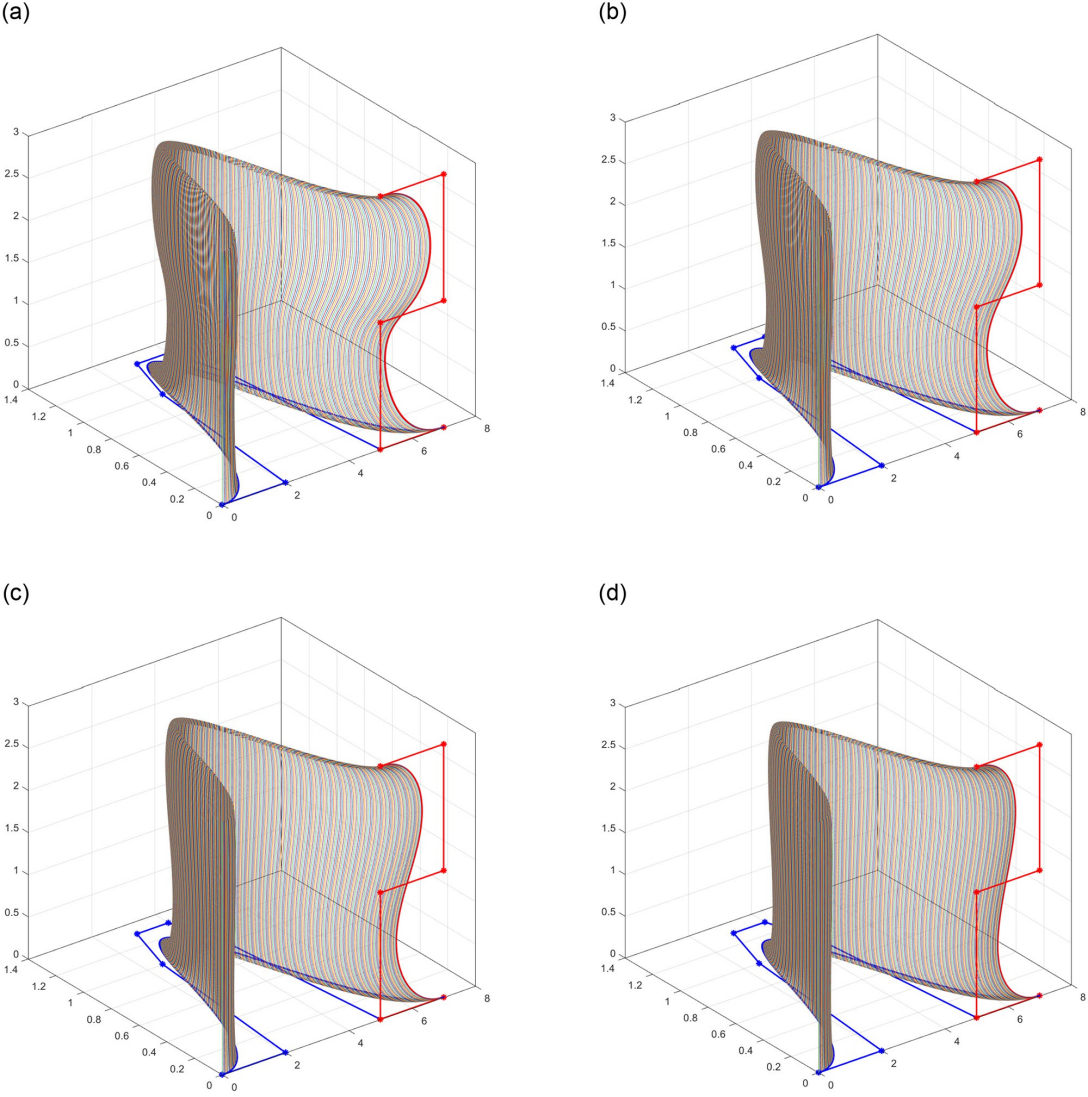
Open swung surface. (a) *l*_1_ = *l*_2_ = −1, (b) *l*_1_ = *l*_2_ = 0, (c) *l*_1_ = *l*_2_ = 1, (d) *l*_1_ = *l*_2_ = 2.

A swung surface with open profile curve of degree 5 and open trajectory curve of degree 6 is constructed using Eq 12 and the results are shown in Fig 7.

### 4.4 Surface of revolution

**Fig 8:**
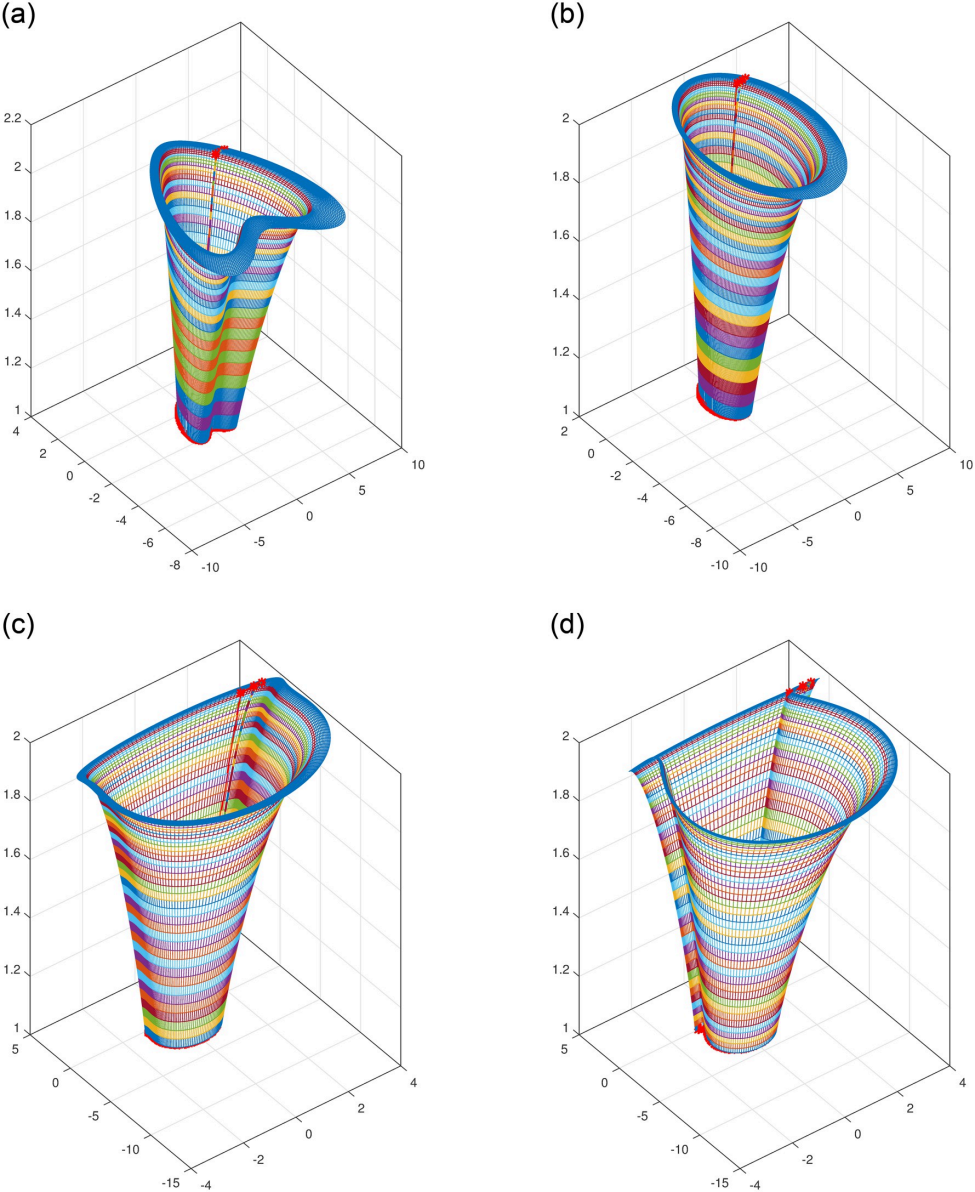
Surface of revolution with different shape parameters. (a) *l*_1_ = *l*_2_ = −1, (b) *l*_1_ = *l*_2_ = 0, (c) *l*_1_ = *l*_2_ = 1, (d) *l*_1_ = *l*_2_ = 2.

A closed sweep surface is also known as surface of revolution and is constructed simply by rotating a 2D curve about any axis, the axis is known as axis of rotation. The surface of revolution is symmetric about the axis of rotation. Taking *γ* = 1, *l* = *m* = 0 and T(v) to be a circle, yields the surface of revolution. Fig 8 shows different surface of revolution with various shape parameters when a curve is rotated in the plane.

### 4.5 Translational surfaces

Translational surfaces can be discussed as a special case of swept surfaces when M(v) is an identity matrix for all v in Eq 13, then C(u) represents a translation of R(v),

$$\begin{array}{c} S(u,v)=R(v)+C(u). \end{array}$$ (14)

**Fig 9:**
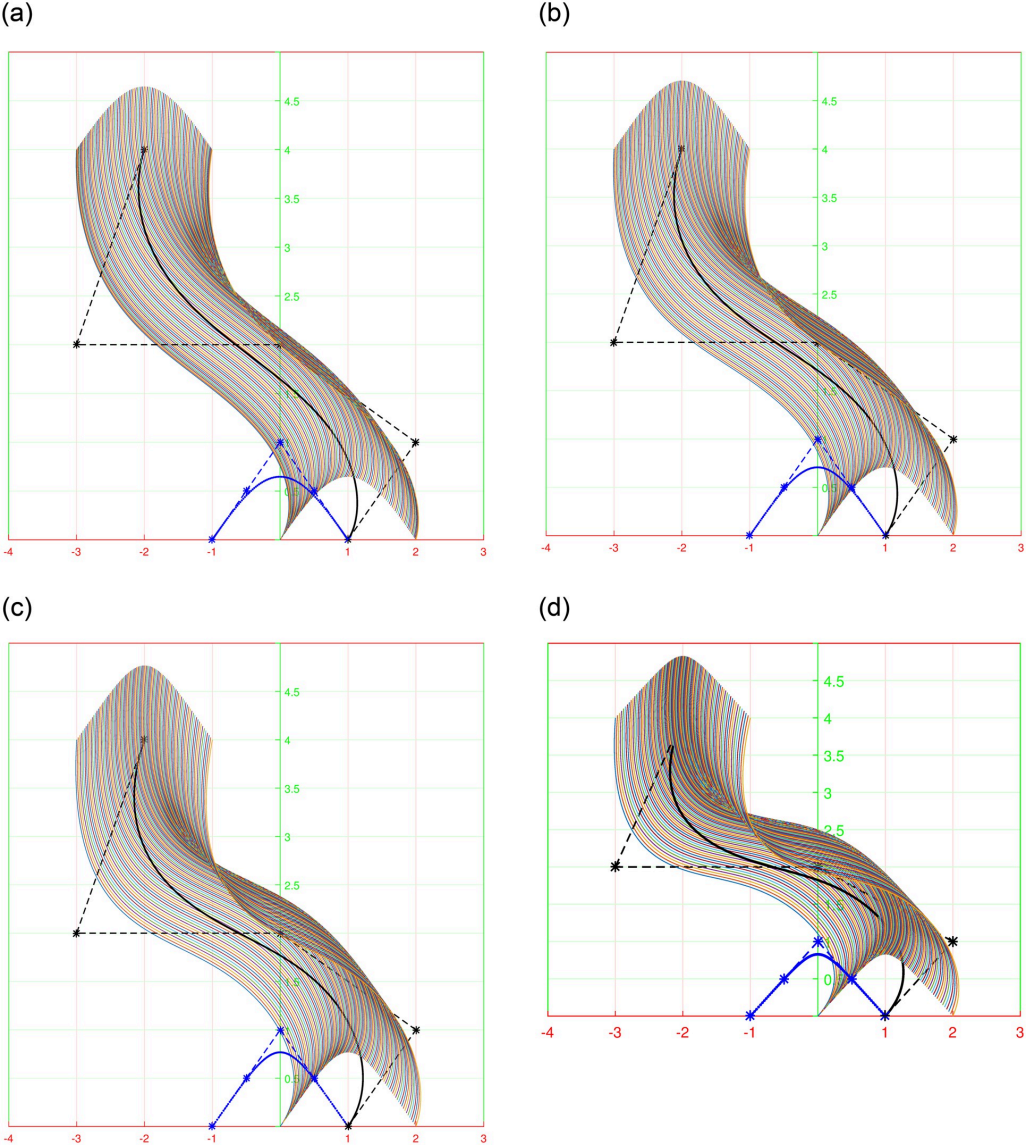
Open translational sweep surfaces with different shape parameters. (a) *l*_1_ = *l*_2_ = −1, (b) *l*_1_ = *l*_2_ = 0, (c) *l*_1_ = *l*_2_ = 1, (d) *l*_1_ = *l*_2_ = 2.

**Fig 10:**
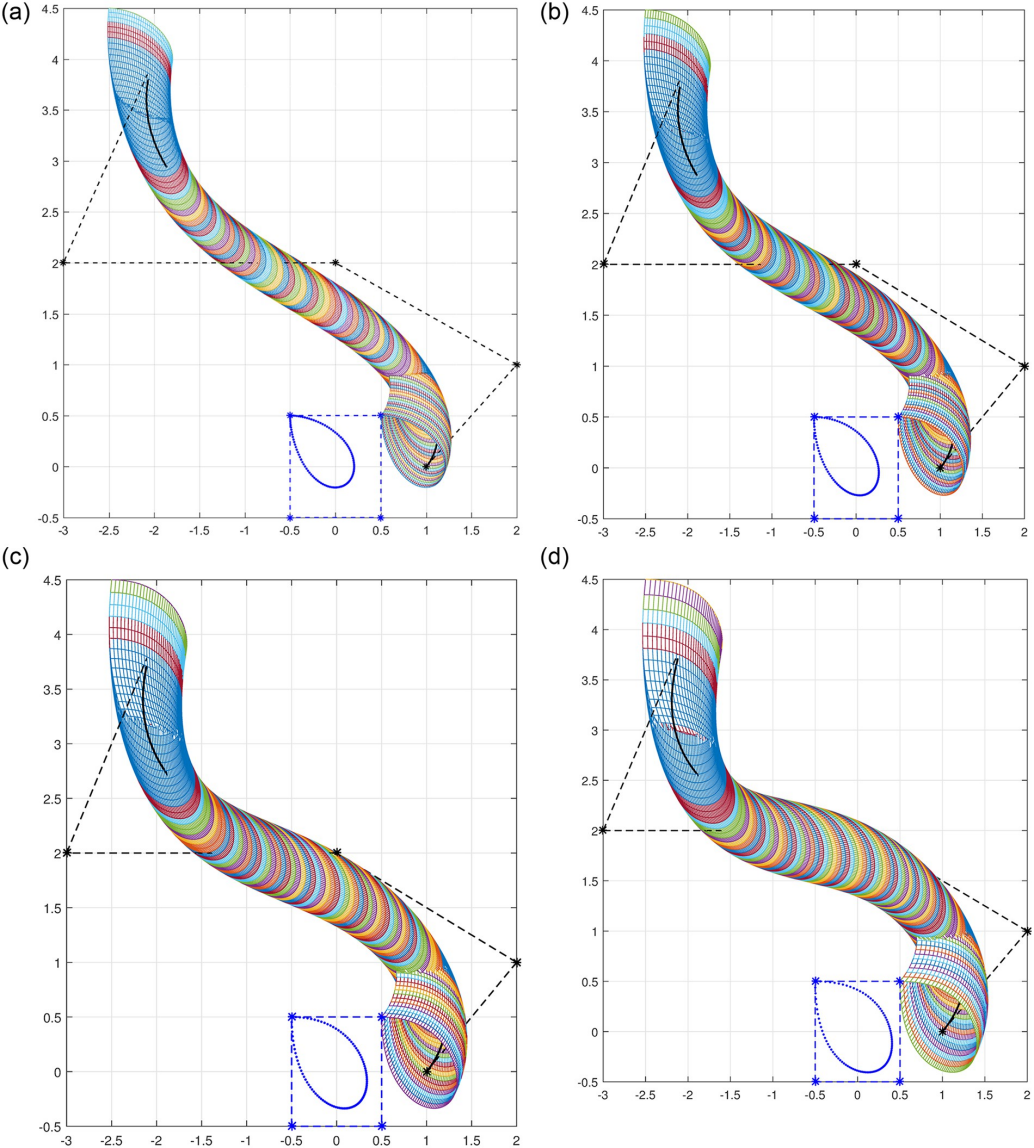
Closed translational sweep surfaces with different shape parameters. (a) *l*_1_ = *l*_2_ = −1, (b) *l*_1_ = *l*_2_ = 0, (c) *l*_1_ = *l*_2_ = 1, (d) *l*_1_ = *l*_2_ = 2.

**Fig 11:**
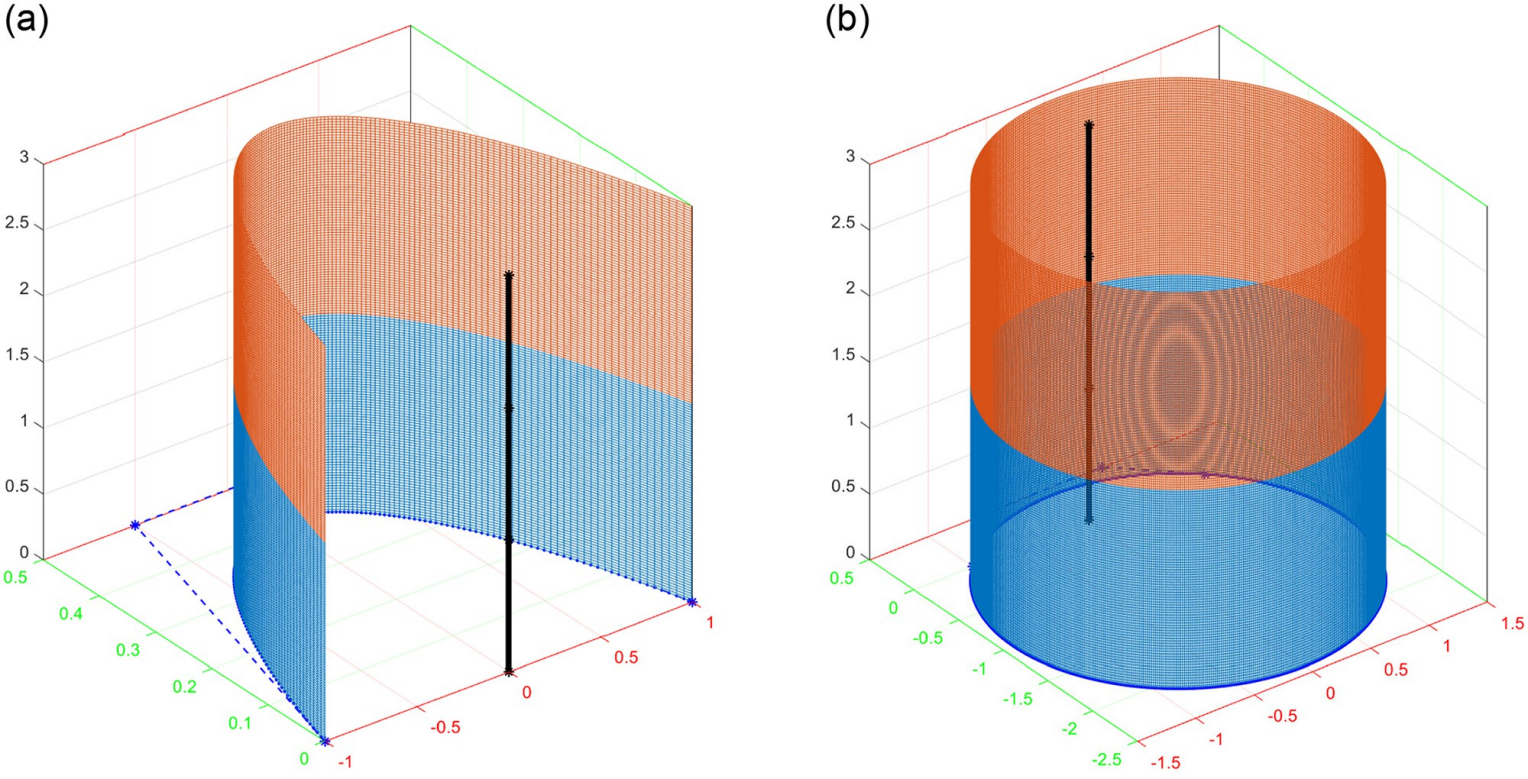
Construction of cylinder for *l*_1_ = *l*_2_ = 1.

This type of surfaces are known as translational sweep surfaces. For illustration an open and a closed sweep surfaces are shown in Figs 9 and 10. Cylinder is a special type of translational surface in which the route curve is a straight line represented in Fig 11.

## 5 Conclusion

In conclusion, the construction of generalized Bézier-like surfaces using newly defined trigonometric polynomial functions with two shape parameters offers a versatile and flexible approach. By incorporating these shape parameters, the resulting surfaces can be adjusted and manipulated to achieve a wide range of desired shapes and characteristics. This allows for enhanced control and customization in surface design. The use of trigonometric polynomial functions provides a rich mathematical foundation that enables the creation of complex and intricate surface geometries. Overall, this approach opens up exciting possibilities for advanced surface construction and opens new avenues for innovation in various fields such as computer-aided design, manufacturing, and visual effects.
